# Latent Gout

**Published:** 1890-02-15

**Authors:** 


### THE PRACTITIONER'S OUTLOOK AND RETROSPECT.

[*The Editor will be glad to receive offers of co-operation and contributions from members of the profession. There is no doubt that much valuable material full of interest and instruction is at present lost to science. This is largely so in the case of* (1) *the younger and less-known members of the hospital staff* (2) *those connected with cottage hospitals, and* (3) *the great body of medical practitioners not attached to any institution. The co-operation of all is cordially invited to enable the Editor to make* THE HOSPITAL *of the utmost possible use and value to the profession. Specialists willing to review books on their special subjects are invited to communicate this fact at once. All letters should be addressed to* THE EDITOR, THE LODGE, PORCHESTER SQUARE, LONDON, W.]

### MEDICINE.

### LATENT GOUT.

Sir William Roberts, formerly Professor of Medicine Victoria University, contributes a paper on Pfeiffer's test for latent gout (*Lancet*, January 4th). The water of the twenty-four hours, after a preliminary filtration through paper, is divided into two portions. One portion is passed through a filter, on which some chemically pure uric acid is laid; an equal volume of both portions is then acidulated with strong hydrochloric acid, and set aside until the uric acid is fully separated. The precipitates from each portion are then collected on weighed filters, duly washed with distilled water, dried, and weighed. Pfeiffer found that in the case of persons not affected with gout, or subject to uric acid gravel, that the urine which had been passed through the uric acid filter yielded nearly as much uric acid as the portion which had not been so passed. In the case of gouty persons, the urine passed through the uric acid filter deposited a large proportion of its uric acid on the filter. From this Pfeiffer concluded that in the gouty urine uric acid exists in a free state, while in those not gouty it existed in combination as a urate. But the results obtained by Pfeiffer's test are varying and inconstant, and depend on the interaction of a number of factors—viz., the degree of acidity of the urine, the comparative richness of the urine in uric acid, the rate at which the filtration is conducted, and the quantity of uric acid placed upon the filter. Something, we think, must also be credited to the freshness or staleness of the urine, and whether it has been kept in corked or uncorked bottles. It appears to be sufficiently proved that there is in gout free uric acid in the urine. But for practical purposes we doubt very much if Pfeiffer's test can come into general use, having to be conducted with much delicacy, and being so liable to error.

